# Extensive range overlap between heliconiine sister species: evidence for sympatric speciation in butterflies?

**DOI:** 10.1186/s12862-015-0420-3

**Published:** 2015-06-30

**Authors:** Neil Rosser, Krzysztof M. Kozak, Albert B. Phillimore, James Mallet

**Affiliations:** Department of Genetics, Evolution and Environment, University College London, Gower Street, London, WC1E 6BT UK; Department of Zoology, University of Cambridge, Downing Street, Cambridge, CB2 3EJ UK; Division of Biology, Imperial College at Silwood Park, Ascot, SL5 7PY UK; Present address: Department of Biology, University of York, Wentworth Way, York, YO10 5DD UK; Present address: Institute of Evolutionary Biology, University of Edinburgh, Ashworth Laboratories, Kings Buildings, West Mains Road, Edinburgh, EH9 3JT UK; Present address: Department of Organismic and Evolutionary Biology, Harvard University, 16 Divinity Avenue, Cambridge, MA 02138 USA

**Keywords:** Geography of speciation, Butterflies, Age-range correlation, Simulations, Ecological speciation

## Abstract

**Background:**

Sympatric speciation is today generally viewed as plausible, and some well-supported examples exist, but its relative contribution to biodiversity remains to be established. We here quantify geographic overlap of sister species of heliconiine butterflies, and use age-range correlations and spatial simulations of the geography of speciation to infer the frequency of sympatric speciation. We also test whether shifts in mimetic wing colour pattern, host plant use and climate niche play a role in speciation, and whether such shifts are associated with sympatry.

**Results:**

Approximately a third of all heliconiine sister species pairs exhibit near complete range overlap, and analyses of the observed patterns of range overlap suggest that sympatric speciation contributes 32 %–95 % of speciation events. Müllerian mimicry colour patterns and host plant choice are highly labile traits that seem to be associated with speciation, but we find no association between shifts in these traits and range overlap. In contrast, climatic niches of sister species are more conserved.

**Conclusions:**

Unlike birds and mammals, sister species of heliconiines are often sympatric and our inferences using the most recent comparative methods suggest that sympatric speciation is common. However, if sister species spread rapidly into sympatry (e.g. due to their similar climatic niches), then assumptions underlying our methods would be violated. Furthermore, although we find some evidence for the role of ecology in speciation, ecological shifts did not show the associations with range overlap expected under sympatric speciation. We delimit species of heliconiines in three different ways, based on “strict and ” “relaxed” biological species concepts (BSC), as well as on a surrogate for the widely-used “diagnostic” version of the phylogenetic species concept (PSC). We show that one reason why more sympatric speciation is inferred in heliconiines than in birds may be due to a different culture of species delimitation in the two groups. To establish whether heliconiines are exceptional will require biogeographic comparative studies for a wider range of animal taxa including many more invertebrates.

**Electronic supplementary material:**

The online version of this article (doi:10.1186/s12862-015-0420-3) contains supplementary material, which is available to authorized users.

## Background

Despite a controversial history, sympatric speciation is now generally accepted as theoretically plausible [1–3] and a good number of examples now exist where it appears a more likely hypothesis than allopatric or parapatric speciation [4–7] (at least under the normal spatial or biogeographic definition of sympatry [8]). Consequently, determining whether sympatric speciation is common or not has become a matter of considerable interest [9]. Contemporary geographical distributions of closely related species, especially the degree of range overlap, have been used to infer dominant modes of speciation at least as far back as the turn of the twentieth century [10] and more sophisticated variants on this approach continue to be used today [11–15].

A popular method for inferring geography of speciation from species distributions is the *age-range correlation*. This approach involves estimating the slope and intercept of the relationship between time since speciation and geographic range overlap. Under allopatric speciation young sister species should have zero overlap, with sympatry between older sister pairs increasing over time as a result of range movements. Conversely, under sympatric speciation the youngest sister species will tend to have sympatric distributions (100 % overlap), while post-speciation range movements may reduce the degree of sympatry of older species-pairs over time [4, 14, 16–20]. The intercept provides a crude estimate of the fraction of speciation that was sympatric. Difficulties arise if a pattern intermediate between these extremes is found, as it may arise either via a single geographic mode of speciation followed by rapid range movements or by a mixture of sympatric and non-sympatric speciation [14, 19–21]. In response to these problems, Phillimore et al. [15] developed spatial simulations of stochastic range change by species following different geographic modes of speciation. In that study, the proportions of sister species showing zero and complete range overlap and their bimodality were found to be more informative than the age-range correlation for estimating the relative frequencies of allopatric and sympatric speciation [16].

A number of studies have inferred the frequency of different geographic modes of speciation, but few have focused on taxa thought to be likely candidates for sympatric speciation [4, 14, 22]. *Heliconius* butterflies and their close allies (Lepidoptera: Nymphalidae: Heliconiini) fulfil certain conditions thought conducive to sympatric speciation. Firstly, wing colour patterns involved in Müllerian mimicry are used also in mate recognition. Furthermore, mate preference colour patterns are often genetically linked to colour pattern loci themselves [23–26]. Reproductive isolation could therefore result from a switch in mimetic pattern in sympatry, since the impediment to divergence that recombination usually poses would be reduced [3, 27]. Speciation may also sometimes be initiated by adaptive introgression of colour patterns between closely related heliconiine species, an inherently sympatric process termed “hybrid speciation” [28–30]. Second, heliconiines are phytophagous, feeding on plants from the family Passifloraceae. In species that exhibit host plant fidelity and mate on their hosts a host plant shift may give rise to reproductive isolation, and indeed several putative cases of sympatric speciation involve phytophagous insects [31–33]. *Heliconius* males frequently patrol host plants and monitor larvae and pupae they find there, with mating often taking place on or near the host [34, 35]. Furthermore, 42 % of *Heliconius* species are known to engage in “pupal mating”, where mating occurs on or near the larval host, before females have fully emerged from pupae [36, 37]. Thus, shifts to new hosts can inhibit gene flow, leading to a build-up of reproductive isolation.

The primary aim of this study is to infer the frequency of sympatric speciation from patterns of range overlap of heliconiine sister species together with phylogenetic branch lengths. We use age-range correlations, and we compare numbers of sister species pairs showing zero or complete range overlap to expectations generated via simulations of random range movements following speciation. For the first time, we also explore the effect of different species concepts on inferences of geographic modes of speciation. To assess whether shifts in mimetic colour pattern, host plant and climatic niche play a role in heliconiine speciation, we examine the relationship between ecological divergence and time since speciation. Finally, we examine the relationship between trait similarity and geographic range overlap.

## Methods

We compiled a database of 58,236 locality records for 76 species and 437 subspecies of heliconiines, in 10 genera (*Agraulis, Dione, Dryadula, Dryas, Eueides, Heliconius, Laparus, Neruda, Philaethria, Podotricha*). We mapped species and subspecies distributions, using α-convex hulls to convert point localities into vector polygons [38] projected in a Lambert Cylindrical Equal Area projection. The dataset and mapping procedure was described in detail earlier [39]. The genus *Philaethria* was not originally included [39] but was mapped for the current study using revised taxonomy and new records [40].

We tested the effects of two versions of the Biological Species Concept (BSC), and conducted a preliminary investigation of one version of the Phylogenetic Species Concept (PSC). Table 1 provides a brief summary of species concepts discussed in this paper.

**Table 1:** Species concepts and their implementation

Species concept	Definition	Practical implementation	Reference
Strict biological	Species are characterised by near complete reproductive isolation.	Semi-species which hybridise freely are lumped.	[41]
Relaxed biological	Species are characterised by substantial but not necessarily complete reproductive isolation.	Semi-species given full species status, c.f. current heliconiine taxonomy.	[42]
Genotypic cluster	Species are delimited by the presence of gaps between clusters of multilocus genotypes within a local area.	Semi-species given full species status.	[44]
Diagnostic phylogenetic	Taxa differing at fixed, diagnostic traits are given species status.	Current heliconiine subspecies raised to species status.	[56]

Under a “strict” interpretation of the BSC, species are groups of interbeeding populations with strong reproductive isolation from other such groups [41]. “Semi-species”, geographic taxa which hybridise at non-trivial rates but show some reproductive isolation at parapatric boundaries, or if allopatric are thought likely to do so if in brought into contact, are lumped within the same species. Under a “relaxed” interpretation of the BSC, species are characterised by substantial but not necessarily complete reproductive isolation [42], with semi-species considered full species, especially if they remain largely distinct in narrow zones of overlap. Relaxed BSC is the current practice in heliconiine taxonomy [39, 43], and relaxed BSC species correspond approximately to those recognised under the Genotypic Cluster Criterion [44]. By way of example, in the well-studied hybrid zone between *H. himera* and *H. erato* hybrids do occur, but are rare compared with the parental forms in areas of overlap [45, 46]. This pair were considered the same species under earlier, stricter versions of the BSC classification of heliconiines (e.g.[47]), but are considered distinct today under a relaxed BSC or GCC because they form a distinguishable pair of genotypic clusters, in spite of ~5 % F1 hybrids in the overlap zone.

**Fig. 1:**
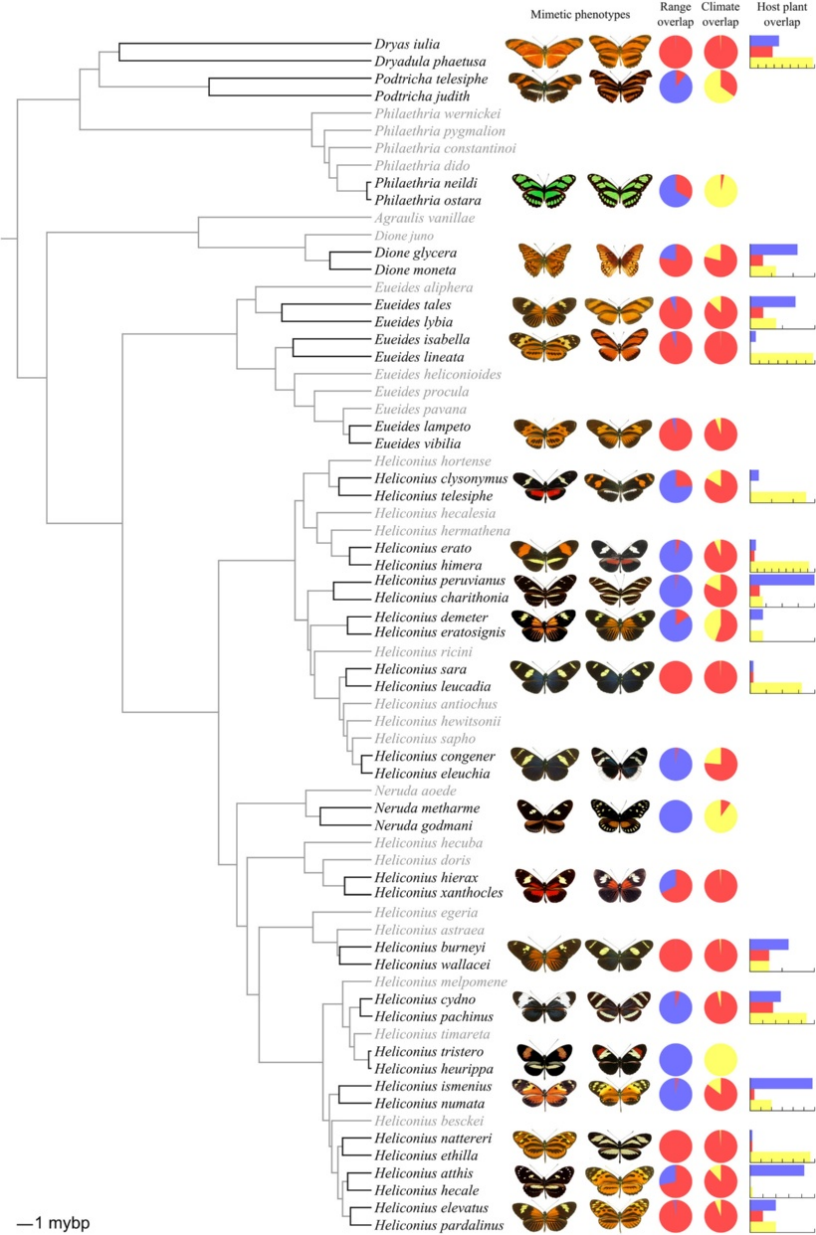
Ultametric Bayesian phylogenetic tree for heliconiines based on 20 nuclear and 2 mitochondrial genes [48], estimated under a relaxed clock model with the age of the split between Heliconiini and Acraeini calibrated following [49]. Sister species pairs are highlighted. Photos show example phenotypes for sister pairs; the upper species in each pair is shown in the photo to the left and the lower species in the photo to the right. Pie charts indicate proportional geographic range and climatic niche overlap; red indicates overlap. Host plant use shows the number of host plant species used by the upper species in blue and the number used by the lower species in yellow, with the number of host plant species shared by the sisters in red. The tick marks on the bar charts represent intervals of 5

Practical implementation of the relaxed BSC follows current taxonomy of heliconiines [39, 40]. Relaxed sister pairs and phylogenetic branch lengths are taken from an ultametric Bayesian phylogenetic tree based on 20 nuclear and 2 mitochondrial genes [48] (Fig. 1; Additional file 1), estimated under a relaxed clock model with the age of the split between Heliconiini and Acraeini calibrated as 47 My [49]. Our results are likely to be robust to phylogenetic uncertainty; earlier analyses conducted using a previously published phylogeny [50] returned results very similar to those presented here [51]. Branch lengths represent millions of years since divergence and were estimated in BEAST v. 1.8 [52] simultaneously with the topology. A strict BSC classification was created by collapsing the phylogenetic nodes of relevant allopatric or parapatric relaxed BSC species (Additional files 2, 3).

Hitherto, no-one has seriously suggested the use of a phylogenetic species concept in heliconiines, and nor do we advocate its use in these taxa. However, it is clear that many heliconiine taxa currently considered subspecies or geographic mimicry races could readily be delimited as species under “diagnostic” versions of the PSC. Some geographic races frequently show fixed differences in mtDNA, as well as colour-pattern traits [53]. It has even been argued on the basis of mitochondrial and nuclear AFLP markers that geographic subspecies in *H. erato*, *H. melpomene* and *H. cydno-timareta* superspecies are “monophyletic” [54] although this is unlikely to be true under most definitions of monophyly. In conclusion, many, perhaps most geographic races in polytypic species of Heliconiini form “irreducible (basal) clusters of organisms, diagnosably distinct from other such clusters, and within which there is a parental pattern of ancestry and descent” [55, 56]. This “diagnostic” version of the PSC specifically allows for the possibility of non-monophyly, and pays no attention to reproductive compatibility, which as a plesiomorphy is regarded as not useful in classification [55].

Phylogenetic branching of heliconiine colour pattern races (our PSC species) is poorly studied except in a few species [53, 54], and in any case would show much reticulation due to abundant gene flow. As a result, we could not carry out analyses using phylogenetic branch lengths, such as age-range correlations. However, we were able to investigate overlap. Recognized heliconiine subspecies [44] are generally monomorphic in at least some part of their range, but some are broadly clinal, especially across the Amazon basin, showing considerable overlap [40]. Probably, these broadly clinal forms would not be classified as separate species by a PSC practitioner. Thus, the PSC species count would be somewhat less than the total numbers of subspecies recognized by today’s lepidopterists, although many more than the number recognized currently.

We quantified overlap between sister species as the area of sympatry divided by the area of the smaller species range, giving an index ranging from 0 to 1 [57]. To account for geographical incompleteness of sampling and small inaccuracies in the mapping procedure, we defined < 0.05 overlap as complete allopatry and > 0.95 overlap as complete sympatry. To avoid subjectivity with the PSC classification, we investigate conspecific overlap for all 427 recognized subspecies, each treated as a separate PSC species. This is equivalent to the assumption that all subspecies within a species are sisters in a star-shaped phylogeny. Our surrogate method provides an upper bound of overlap shown by PSC species with their sister species. Based on better phylogenetic information, overlap of each suitable species with at most one other PSC (its sister-species) would be calculated, and would presumably be less than the overlap values used here. Similarly, the fraction showing complete allopatry would therefore be higher.

### Age-range correlations

We tested for an age-range correlation, using ordinary least squares regression with the angular-transformed degree of sympatry between species pairs as the dependent variable, and molecular phylogenetic branch length as the predictor. Unlike several previous analyses, we included only sister species in the regression, thus avoiding the problem of reconstructing ancestral ranges for comparisons within the phylogeny [58].

### Simulations

The simulation-based approach employed broadly follows Phillimore et al. [15]. Here, we give a brief outline. Each replicate set of simulations modelled speciation and the subsequent range shifts of the daughter species as many times as there are pairs of sister species. Speciation was simulated by dividing the area of an ancestral geographic range into two square daughter ranges, whose positions and sizes relative to one another depended on the mode of speciation being employed (allopatric, peripatric, parapatric or sympatric; details below). The total area available was a square grid of 100 × 100 units. We used heliconiine species range sizes to set the size of the initial ancestral range. We defined the area available to heliconiines as the total area occupied by the tribe, and calculated the median range size of all heliconiine species relative to the total area occupied by the tribe (8.2 % under a relaxed BSC, 13.5 % under a strict BSC) and the median range size of all sister species relative to the total area occupied by the tribe (10.8 % relaxed BSC, 16 % strict BSC). We then used these percentages as the size of the ancestral range relative to the square grid in simulations. We also ran simulations with ancestral ranges of double these sizes (because the simulations start by dividing the ancestral range in two).

When simulating sympatric speciation between a sister pair, the ancestral range was randomly divided in two, with the smaller daughter range then placed randomly within the larger daughter range. For simulations of non-sympatric speciation, we varied the geographic configurations of the ranges to be either vicariant, peripatric and parapatric. We simulated vicariant allopatric speciation by randomly dividing the ancestral range and placing the larger of the two ranges randomly on the grid, with the smaller species range then placed 2 grid squares to the right of the first, but with its position on the vertical axis permitted at any point along the vertical extent of the larger range. In simulations of peripatric speciation, the ancestral range was split into two unequally sized parts using the ratio 95:5, and the two ranges were then placed randomly on the grid with the constraint that they could not overlap initially. Parapatric speciation was simulated in the same way as vicariant allopatric speciation, except that the species ranges abutted.

Post-speciation range movements were simulated by adding a random normal deviate with a mean of 0 to the vectors corresponding to the top, bottom, right and left extents of each species range. This process was repeated at each time step. Thus although species ranges started as squares, they could become rectangular over the course of a simulation. Different rates of range change were explored by varying the standard deviation of distribution from which the values were drawn, we used 0.25, 0.5, 0.75, 1, 1.5, and 2. We also examined the effect of giving the species ranges a tendency to grow by increasing the mean of the standard normal deviate to 0.1. We parameterised the duration of simulations using the phylogenetic branch lengths of sister species as relative estimates of time since speciation. We did this by randomly assigning (without replacement) the branch lengths to each simulated sister species pair. The number of time steps for each simulated sister pair was then generated by multiplying the assigned branch length value by 10. We did not vary the value of the multiplier due to redundancy with the range movement parameter, i.e. varying the rate of range change has a similar effect to varying the time length of simulations. If one of the daughter species dwindled to zero range size then the simulation was repeated until it resulted in two surviving species.

We ran 1000 replicates for each possible combination of parameters and explored all possible proportions of sympatric speciation events. For the observed heliconiine data and for each replicate set of simulations we calculated three indices: the numbers of sister pairs exhibiting (i) complete range overlap, (ii) zero range overlap and (iii) the degree of bimodality of the overlap distributions. Bimodality of data was quantified in the range 0–1, as (*z* x *c*)/(*a* x *b*), where *z* and *c* are the number of cases of complete sympatry and allopatry, and *a* and *b* are the number of cases of complete sympatry and allopatry that would occur if all the data were split evenly between these states [15]. We then compared the indices calculated for heliconiines with those generated via simulations using a two-tailed test. Simulation parameters were considered to be unlikely to give rise to the observed heliconiine data if the observed values fell outside the 2.5 and 97.5 percentiles of the simulated distribution.

### Ecological divergence

Following a relaxed BSC, we classified heliconiine species wing colour patterns (Additional file 4) using an updated and modified version of a published colour pattern scheme [47, 59]. This scheme classifies colour patterns into broad mimicry rings (e.g. black with yellow forewing band and red hind-wing band). These mimicry rings may be further subdivided, but speciation via colour pattern shift seems more likely to be driven by major shifts rather than minor variations [23]. Host plant records were obtained from a compilation [60]. We excluded all records marked as dubious, and all those known/thought to have been recorded from captive populations. We also excluded all records where the host plant identification was marked as doubtful. If a host plant was identified as similar (but distinct) to a known species, it was treated as a separate species. We measured the similarity of colour patterns and host plants between sister species as x/y, where x is the number of mimetic patterns or host plants shared, and y is the total number used by the sister that has fewer mimetic patterns or uses fewer host plants.

To quantify divergence in climatic niche, we downloaded the 19 bioclimatic variables available on the Worldclim website, at 10 arc-minute resolution. We clipped the rasters to include only the Americas between 48° north and 36.6° south, which is the latitudinal range for which heliconiines have been recorded. We carried out a Principal Components Analysis (using the correlation matrix) to reduce the bioclimatic variables to 3 principal components, which explained 84 % of the variation. We then plotted heliconiine species in 3-dimensional niche space (each dimension corresponds to a principal component) using the program NicheA (H. Qiao, J. Soberón, L. Campbell & A. Townsend Peterson, available at http://biodiversity-informatics-training.org/software-data-sources/nichea). We estimated a species’ climatic niche as the minimum convex polyhedron encompassing all the data for a species. Overlap between species pairs was calculated as x/y, where x is the area of the intersection between the two polyhedra, and y is the area of the smaller of the two polyhedra.

To investigate whether shifts in ecology are associated with speciation, we tested whether the degree of similarity in mimicry, host plant and climatic niche overlap between sister species is predicted by the time that has elapsed since they shared a common ancestor (measured as molecular phylogenetic branch length). We interpret the intercept as an estimate of trait similarity at speciation, with the slope indicative of the general trend of trait divergence or convergence. If ecological shifts are associated with speciation we expect a low intercept, indicating low sharing of traits at speciation. For mimetic and host plant overlap, we tested this using a generalised linear model with binomial errors. When we detected overdispersion we corrected the standard errors using a quasi-GLM model where the variance is given by *ϕ*μ, where μ is the mean and *ϕ* is the dispersion parameter. For climatic niche overlap, we used simple linear regression with angular-transformed niche overlap values as the response. Finally, we used multiple regression to explore the relationship between trait divergence and angular-transformed geographic range overlap.

## Results

### Observed range overlap of heliconiines

**Fig. 2:**
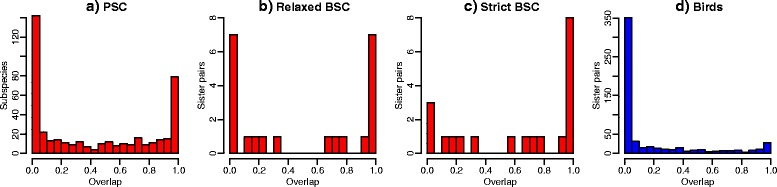
Histograms of range overlap for heliconiine butterflies and birds. **a** diagnostic phylogenetic species (PSC), (subspecies used as a surrogate for PSC species), **b** relaxed biological species (BSC) sister species, **c** strict BSC sister species. For comparison, in **d** we show range overlap for sister species of birds [16]

**Fig. 3:**
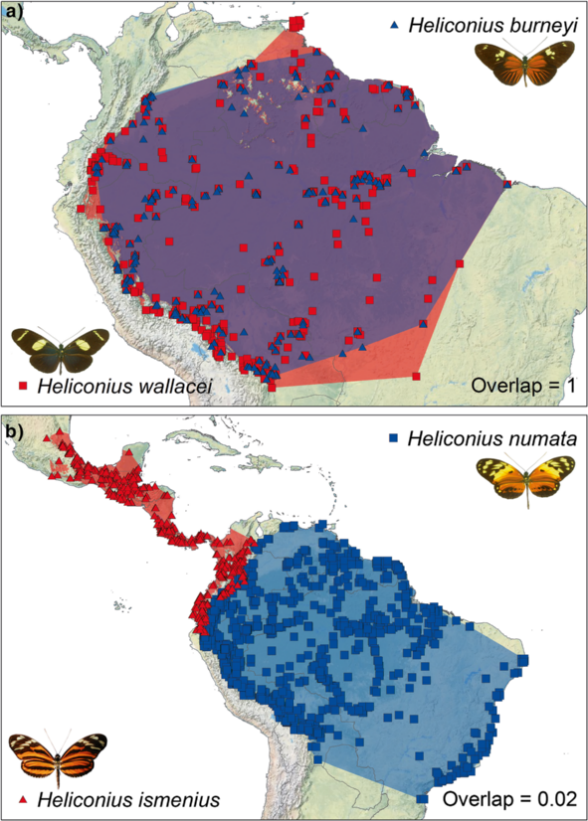
Examples of **a** an overlapping and **b** a non-overlapping pair of sister species [39]

Heliconiine sister species overlapped completely in 7 out of 22 cases under a relaxed BSC and 8 out of 20 cases under a strict BSC (Fig. 2). Examples of overlapping and non-overlapping relaxed BSC sister species ranges are shown in Fig. 3. The number of non-overlapping sister species was more strongly influenced by species concept, with 7 out of 22 cases under a relaxed BSC and only 3 out of 20 cases under a strict BSC. Accordingly, the bimodality score for overlap was higher under a relaxed BSC (0.40) than under a strict BSC (0.24). In contrast to these results for BSC species, when heliconiine subspecies were delimited as phylogenetic species (PSC), far more complete allopatry (142 cases out of 427 cases) was found than complete overlap (79 cases), with a bimodality score of 0.74. Nonetheless, there is still some complete overlap (19 %) due to the clinal nature of some taxa. Because our surrogate PSC overlap measure measures overlap with all other PSCs that are members of the same relaxed BSC, actual overlap among only sister PSC species would be even lower. Our estimates of PSC overlap give upper bounds. Nonetheless, it is clear that a PSC classification of heliconiines would result in even lower levels of overlap, implying higher levels of allopatric speciation than under BSC classifications (Fig. 2).

### Age-range correlations

**Fig. 4:**
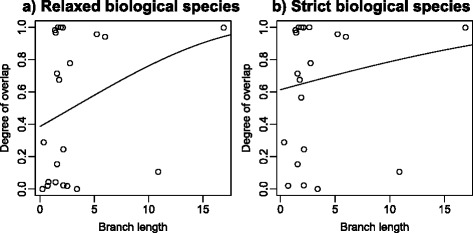
Age-range correlations for **a** relaxed and **b** strict biological species. Branch lengths represent millions of years since divergence. Linear regressions were performed on angular-transformed degree of overlap, with the data then back-transformed for plotting

Using a relaxed BSC (Fig. 4a) we estimated an intercept of 0.67 ± 0.16 and slope of 0.04 ± 0.03 (*P* = 0.26, back-transformed intercept = 0.39). Under a strict BSC (Fig. 4b) we estimated an intercept of 0.9 ± 0.17 and slope of 0.02 ± 0.03 (*P* = 0.57, back-transformed intercept = 0.61). Lack of phylogenetic branch lengths precludes obtaining a similar estimates for PSC species.

### Comparison of the heliconiine data with simulation results

**Fig. 5:**
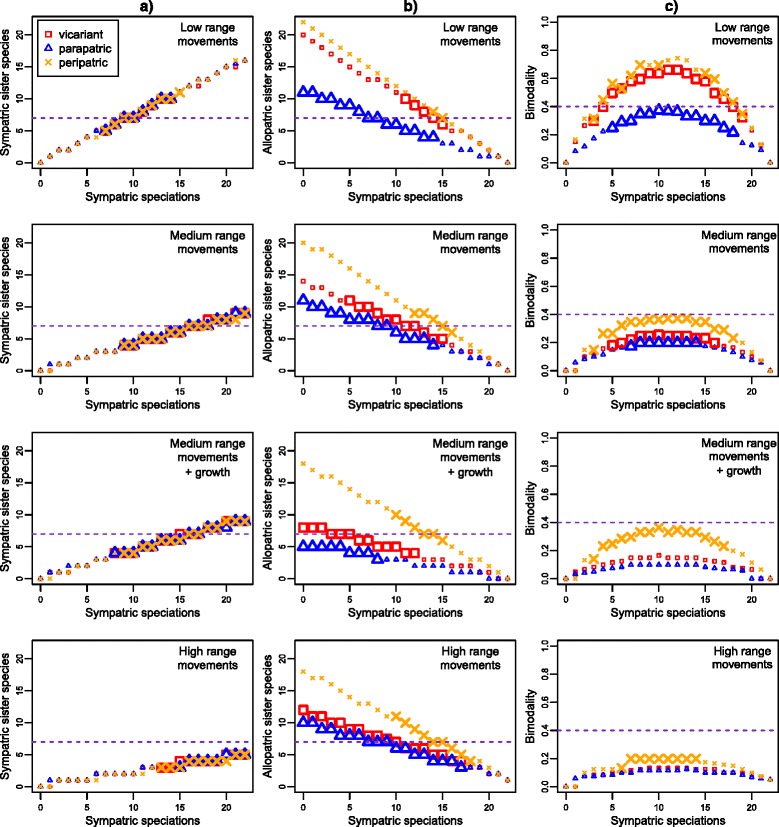
Examples of simulation results for relaxed biological species using selected parameters. The x-axis shows the number of sympatric speciation events, the y-axis shows the median number of completely sympatric species (column **a**), the median number of allopatric species (column **b**) and the median bimodality (column **c**). The dotted purple lines show the values observed for heliconiines. Simulations were run using medium-sized starting ranges (the size of the ancestral ranges relative to the total area available =16 %), while varying the rate of range movements and the tendency of ranges to grow following speciation. The geographic configuration of the allopatric range (vicariant, parapatric or peripatric) is shown in the key. Simulations results that were not significantly different from the observed values are indicated with bold symbols

In simulations where most speciation was allopatric or parapatric, completely overlapping sister species were rare (Fig. 5a). This is because even extensive random range movements rarely caused sister species originating in allopatry to become completely sympatric. This is true even when species ranges are given a tendency to grow following speciation. In contrast, the number of cases of non-overlapping sister species was highly variable, because even small range movements after allopatric speciation can easily lead to some limited geographic overlap between sister species (Fig. 5b).

In simulations where most speciation was sympatric, non-overlapping sister species were always rare. This is because very extensive range movements are necessary to make species ranges that arose in sympatry entirely allopatric (Fig. 5b). However, the number of cases of completely overlapping sister species was highly variable, because even small range movements will often move species ranges out of complete range overlap (Fig. 5a).

The observed number of completely overlapping heliconiine sister species pairs was unlikely (*P* < 0.05 under a two-tailed test) to arise when sympatric speciation comprised less than 32 % (relaxed BSC) or 40 % (strict BSC) of all speciation events. The observed number of non-overlapping pairs was less useful in discriminating geographic speciation scenarios. Under a relaxed BSC this observation is unlikely only when all speciation was sympatric (i.e. 22/22 sister pairs). Under a strict BSC it arose in simulations with all proportions of sympatric speciation (although not under all combinations of parameters). The bimodality scores of the observed data were unlikely to arise in simulations where either allo/parapatric or sympatric speciation predominated (Fig. 5c), and are consistent with simulations where 18–86 % of speciation was sympatric under a relaxed BSC, and 0–95 % under a strict BSC.

In summary, the simulations that were consistent (P ≥ 0.05) with the observed data for all three indices had frequencies of sympatric speciation between 40 %–95 % (strict BSC), 32 %–77 % (relaxed BSC). Still lower levels of sympatric speciation would have been inferred due to lower levels of overlap for the PSC (not tested). These results were obtained with low to high range movements (0.25-2), either zero or positive range growth, and with non-sympatric speciation events that were vicariant, parapatric or peripatric. All ancestral range sizes were able to generate the observed data. However, when starting range sizes covered a larger proportion of the simulation domain area, the observed data arose (*P* ≥ 0.05) under a wider range of conditions.

### Trait divergence

**Fig. 6:**
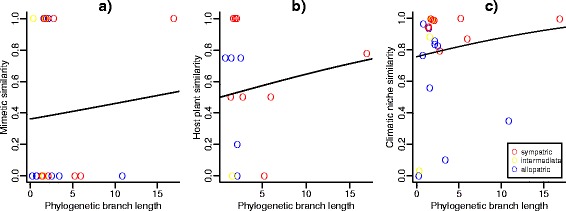
Similarity in aposematic wing pattern (**a**), host plant use (**b**) and climatic niches (**c**) between sister species, plotted against phylogenetic branch length. Colours represent the degree of range overlap between the sisters: blue = range overlap < 0.05, red = range overlap > 0.95, with yellow indicating intermediate levels of range overlap)

14 of 23 sister species pairs have no colour patterns in common (Additional file 4). We estimated a positive but non-significant slope for the relationship between sister species’ colour pattern similarity and phylogenetic branch length (Fig. 6a; *β*_1_ = 0.04 ± 0.12, values on logit scale) and an intermediate intercept (*β*_0_ = -0.57 ± 0.6 on logit scale, or 0.36 as a proportion with 95 % confidence intervals from 0.14 to 0.64). Although none of the heliconiine sister species with available data have been recorded feeding on exactly the same set of host plant species, three species use a subset of the host plants recorded for their sister. Five sister pairs show no overlap in host plants, with the remaining sister pairs showing varying degrees of overlap. For the relationship between sister species’ host plant similarity and phylogenetic branch length, we estimated a positive but non-significant slope (Fig. 6b; *β*_1_ = 0.06 ± 0.05, values on logit scale) and an intermediate intercept (*β*_0_ = -0.01 ± 0.42 on logit scale, or 0.5 as a proportion with 95 % confidence intervals from 0.30 to 0.70). Most sister species have similar climatic niches (77 % of species pairs have overlap values > 0.75). For the relationship between sister species’ climatic niche similarity and phylogenetic branch length (Fig. 6c), we estimated a positive but non-significant slope (*β* = 0.02 ± 0.03, values on an arc sine scale) and a high intercept (*α* = 1.05 ± 0.12 on an arc sine scale, or 0.76 as a proportion with 95 % confidence intervals from 0.5 to 0.93). Multiple regression found no association between ecological similarity and geographic range overlap (Table 2).

**Table 2:** Model coefficients for multiple regression of ecological similarity and phylogenetic branch length against angular transformed geographic range overlap

	Coefficient	SE	t value	*P* value
Intercept	−1.88	1.37	−1.37	0.20
Branch length	0.03	0.04	0.65	0.53
Mimicry overlap	0.33	0.34	0.97	0.35
Host plant overlap	−0.22	0.44	−0.49	0.64
Climatic niche overlap	3.02	1.62	1.86	0.09

## Discussion

We found that 32–40 % of heliconiine sister species show complete (>0.95) range overlap and 50–65 % have range overlap > 0.5, depending on whether a relaxed or strict biological species concept is employed. Only if we classify heliconiine subspecies as full species, under an extreme version of the diagnostic version of the phylogenetic species concept, is the percentage of complete overlap estimated to be less than 19 %. These findings are in strong contrast to a global analysis of bird species, where only 5 % of sister species show complete overlap [15] – see Fig. 2. The Neotropical ovenbirds, which have similar divergence times and geographic distributions to heliconiines, also show very little range overlap: 71.3 % of sisters are allopatric as defined here, with only 3.2 % sympatric [61]. In mammals, sympatric sister species are relatively rare: 14–23 % show overlap > 0.5, although some neotropical groups such as the cats do show considerable overlap [19]. The intercepts for the age-range correlations taken at face value suggest that 39 % (relaxed BSC) or 61 % (strict BSC) of speciation events are sympatric in heliconiines. However, these intermediate intercepts could also have arisen due to rapid range movements since speciation [14, 21], and the high proportion of completely sympatric sister species we observed in heliconiines may be more informative about the process of speciation [15]. According to simulations, the high proportions of entirely sympatric sister species are unlikely to arise without sympatric speciation making a substantial contribution, even if range movements are extensive. Observed patterns of overlap for the BSC classifications were most consistent with models in which sympatric speciation is common, contributing between 32 % and 95 % of all speciation events.

Our inference of a high frequency of sympatric speciation seems exceptional in comparison with analyses of other taxa. However, recent genomic work has uncovered a case where at least 40 % of the genome has been exchanged between sympatric populations due to occasional hybridization by a pair of species; *H. cydno* and *H. melpomene* [62] that would be considered sister species under a stricter BSC classification (where *H. cydno* would be united with *H. timareta*, *H. heurippa* and other allopatric forms). This introgression has been on-going since soon after speciation, indicating long-term sympatry between the two. Pleiotropy and linkage between colour pattern and mate preference in *Heliconius*, along with their phytophagous habits, also support the plausibility of a high rate of sympatric speciation [24–26]. Moreover, some *Heliconius* species appear to have arisen as a result of adaptive introgression of colour patterns [28, 29, 63, 64]. This hybrid speciation necessarily requires at least some degree of sympatry between the parent species and their offspring. Our analyses of mimetic and host plant divergence against phylogenetic branch length generated intermediate intercepts, which could have a number of interpretations. In the case of mimicry shifts, it seems most likely that the intermediate intercept is the result of a mixture of speciation modes (i.e. speciation sometimes occurs with a mimicry shift, and sometimes without). In the case of host plant divergence, the intermediate intercept may indicate that speciation occurs with varying degrees of host plant differentiation, ranging from zero to complete overlap in host plant use. Both plots suggest that the mimicry and host plant differences are not simply the result of gradual divergence over time, and may at least sometimes be associated with speciation.

Some simplistic assumptions of our simulations are that species ranges move independently and stochastically following speciation, and at the same mean rate across all species. These assumptions are suspect, since they ignore the effects of competition, which should be especially important in sympatric speciation and indeed in any model of species coexistence. For instance, closely related species may be ecologically similar due to their common ancestry, with the result that competition prevents them from entering sympatry [65]. On the other hand, if speciation involves a shift in host plant or some other ecological dimension, competition between sister-species would be reduced, and overlap could be favoured. Because climatic niches are likely to be conserved in a new pair of sister species [66], this could rapidly lead to geographic distributions that broadly overlap, irrespective of the geography of speciation. Indeed, our comparative analysis shows climatic niches to be more conserved between sisters than either mimicry or host plants (Fig. 6). We did not, however, find any relationship between range overlap and mimetic or host plant similarity, although this was expected both under sympatric speciation and under the range expansion hypotheses, and also simply due to competition. The lack of a correlation may stem from unmeasured facets of heliconiine niches. For example, some *Heliconius* are known to use different host plants throughout their range [67], details which are hidden in the present analysis due to insufficient data being available to map such patterns. It is also possible that heliconiine densities may be regulated by factors other than direct competition for host plants, such as larval predators and parasites, but these aspects of *Heliconius* ecology are poorly known. Nonetheless, in some species host plants are definitely limiting and competition appears important in preventing spatial overlap [68].

In summary, we cannot entirely rule out the possibility that the initial stages of speciation may involve parapatric or allopatric divergence of a population (as, for example, in the refugium hypothesis [69]), followed by rapid range expansion and geographic overlap between sister species. The differences in overlap between birds and butterflies would then depend more on the ecological drivers of speciation and coexistence than on the geographic milieu of speciation. For example, ecological niches in birds might rarely be as specialized as host plant use in butterflies. Interestingly, two of the most compelling examples of sympatric, ecological speciation in birds involve host-specific crossbills [70] and brood parasitic *Vidua* finches [5].

Our results are dependent on the species concept employed, with higher inferences for the frequency of sympatric speciation obtained using a strict version of the biological species concept than a relaxed version. Lower frequencies of sympatric speciation would be estimated, according to our surrogate method, if heliconiines were classified according to the diagnostic version of the phylogenetic species concept. Under the diagnostic version of the PSC, geographic variants that show “a parental pattern of ancestry and descent” are considered full species. In the relaxed BSC, partially isolated “semi-species” are considered full species, while other geographic taxa that hybridize freely are not. Finally, in the strictest version of the BSC, all geographic variants, including occasionally hybridizing semi-species are considered conspecific. Differences in taxonomic practice may therefore go some way towards explaining why birds (for which the diagnostic phylogenetic species concept was originally developed) display patterns of sister-species overlap so different from those of heliconiines.

## Conclusions

Closely related animal species (in particular vertebrates) often have allopatric distributions (“Jordan’s Law”), and this observation has militated against sympatric speciation [10, 42, 71–74]. Modern phylogenetic methods coupled with increasing availability of spatial biodiversity data now allow such patterns to be tested for a wider array of taxa than was previously possible. In heliconiine butterflies, sister species tend to disobey Jordan’s Law; they are commonly sympatric. Using methods previously suggesting low rates of sympatric speciation in birds and mammals, we infer high rates of sympatric speciation in heliconiine butterflies. Furthermore, heliconiines possess genetic and ecological characteristics that can circumvent theoretical difficulties facing sympatric speciation [42]. Given available comparative and ecological data, therefore, sympatric speciation may well occur in heliconiines. Unfortunately, we cannot entirely rule out the possibility that speciation occurs mainly in allopatry or parapatry, but that rapid range expansion following speciation leads to a high degree of range overlap among sister species.

Critical evidence for sympatric vs. allopatric speciation in heliconiines might be obtained by studying more examples along the speciation continuum, from local polymorphs to sympatric species exhibiting near complete assortative mating. Heliconiines are typically locally monomorphic in colour pattern, but mimicry polymorphisms do exist [75, 76], in one case with weak assortative mating [25], suggesting the beginnings of a process of sympatric speciation. Sympatric species with strong, but incomplete assortative mating are also known; in one population of the largely sympatric species pair *H. cydno* and *H. melpomene* in San Cristóbal, Venezuela up to 8 % of individuals are hybrids [28]. Thus, much rests on identifying and studying gene flow and divergent selection in sympatric taxa exhibiting intermediate levels of assortative mating that fall in the middle of the speciation continuum.

## Additional files

Additional file 1:
**Relaxed biological species sister comparisons, with associated branch length and range overlap values.**
Additional file 2:
**Strict biological species sister comparisons, where different from Relaxed biological species sister comparisons.**
Additional file 3:
**Strict biological species sister comparisons, with associated branch length and range overlap values.**
Additional file 4:
**Colour pattern classification for heliconiines [** 3 **].**
