# Supplementary material for: Extensive range overlap between heliconiine sister species: evidence for sympatric speciation in butterflies?
Source: BMC Evol Biol. 2015 Jun 30;15:125. doi: 10.1186/s12862-015-0420-3 (PMC4486711; doi:10.1186/s12862-015-0420-3)
Supplement: Additional file 2: — Strict biological species sister comparisons, where different from Relaxed biological species sister comparisons. [file 12862_2015_420_MOESM2_ESM.docx]

Strict biological species (where different from [1, 2])

| Strict sister species | Synonyms |
| --- | --- |
| *Heliconius charithonia* | *Heliconius peruvianus* |
| *Heliconius clysonymus* | *Heliconius hortense* |
| *Heliconius cydno* | *Heliconius heurippa* |
|  | *Heliconius pachinus* |
|  | *Heliconius timareta* |
|  | *Heliconius tristero* |
| *Heliconius elevatus* | *Heliconius luciana* |
| *Heliconius erato* | *Heliconius chestertonii* |
|  | *Heliconius himera* |
| *Heliconius sapho* | *Heliconius hewitsoni* |

## References

1. Rosser N, Phillimore AB, Huertas B, Willmott KR, Mallet J: Testing historical explanations for gradients in species richness in heliconiine butterflies of tropical America. *Biol J Linn Soc* 2012, 105:479–497.

2. Constantino LM, Salazar JA: A review of the Philaethria dido species complex (Lepidoptera: Nymphalidae: Heliconiinae) and description of three new sibling species from Colombia and Venezuela. *Zootaxa* 2010, 2720:1–27.
