# Supplementary material for: Extensive range overlap between heliconiine sister species: evidence for sympatric speciation in butterflies?
Source: BMC Evol Biol. 2015 Jun 30;15:125. doi: 10.1186/s12862-015-0420-3 (PMC4486711; doi:10.1186/s12862-015-0420-3)
Supplement: Additional file 3: — Strict biological species sister comparisons, with associated branch length and range overlap values. [file 12862_2015_420_MOESM3_ESM.docx]

Strict biological species sister comparisons, with associated branch length and range overlap values. Branch lengths represent millions of years since divergence.

| **Sister species comparison** | **branch length** | **range overlap** |
| --- | --- | --- |
| *Dione glycera* vs. *Dione moneta* | 2.76 | 0.78 |
| *Dryas iulia* vs. *Dryas phaetusa* | 16.91 | 1.00 |
| *Eueides isabella* vs. *Eueides lineata* | 5.23 | 0.96 |
| *Eueides lampeto* vs. *Eueides vibilia* | 1.44 | 0.97 |
| *Eueides lybia vs. Eueides tales* | 5.98 | 0.94 |
| *Heliconius atthis* vs. *Heliconius hecale* | 1.56 | 0.71 |
| *Heliconius burneyi* vs. *Heliconius wallacei* | 2.10 | 1.00 |
| *Heliconius clysonymus* vs. *Heliconius telesiphe* | 2.15 | 0.25 |
| *Heliconius congener* vs. *Heliconius eleuchia* | 0.71 | 0.02 |
| *Heliconius cydno* vs. *Heliconius melpomene* | 1.90 | 0.57 |
| *Heliconius demeter* vs. *Heliconius eratosignis* | 1.57 | 0.15 |
| *Heliconius elevatus vs. Heliconius pardalinus* | 1.38 | 0.98 |
| *Heliconius erato vs. Heliconius hermathena* | 2.65 | 1.00 |
| *Heliconius ethilla vs. Heliconius nattereri* | 1.91 | 1.00 |
| *Heliconius hierax* vs. *Heliconius xanthocles* | 1.76 | 0.68 |
| *Heliconius ismenius* vs. *Heliconius numata* | 2.14 | 0.02 |
| *Heliconius leucadia vs. Heliconius sara* | 1.66 | 1.00 |
| *Neruda godmani vs. Neruda metharme* | 3.40 | 0.00 |
| *Philaethria neildi* vs. *Philaethria ostara* | 0.34 | 0.29 |
| *Podotricha judith vs. Podotricha telesiphe* | 10.88 | 0.11 |
